# Bacteriophage-mediated gut microbiota regulation: a bibliometric landscape analysis (2005–2024)

**DOI:** 10.3389/fmicb.2026.1768117

**Published:** 2026-03-06

**Authors:** Jiantao Yin, Hefei Wang, Yang Cui, Xinyu Zhou, Shuo Zhang, Hongna Yin

**Affiliations:** 1 The Second Affiliated Hospital of Heilongjiang University of Traditional Chinese Medicine, Harbin, China; 2 Heilongjiang University of Chinese Medicine, Harbin, China

**Keywords:** bacteria, bacteriophage, bibliometric analysis, gut microbiota, host health

## Abstract

**Objective:**

This study systematically evaluates the current situation, knowledge structure and development trend of bacteriophage-mediated intestinal microbiota regulation research from 2005 to 2024 through literature measurement analysis.

**Method:**

Retrieve relevant research from the Web of Science core collection, Scopus and PubMed databases. After screening according to inclusion criteria, CiteSpace, VOSviewer, and R-bibliometrix were employed for bibliometric and visualization analysis. This systematically mapped publication trends, collaboration networks among countries/institutions/authors, core journals, and research hotspots.

**Results:**

Thousands of relevant studies were included. From 2005 to 2024, the number of papers published in microbiology journals showed a step-by-step increase, reaching a peak of 355 articles in 2022. The United States and China are the main contributors. University College Cork in Ireland and San Diego State University in the United States have become core research institutions, and Colin Hill is listed as the most influential author. The Frontiers in Microbiology has published the largest number of papers, and Microbiome and Nature Communications have become platforms for the publication of high-impact research results. The research focus has shifted from the description of early bacteriophage-bacterial binary interaction to exploring the ternary relationship of “bacteriophage-microbiota-host health.” In recent years, short-chain fatty acids, microbiota disorders and clinical intervention have become the core research directions. This study included 16 clinical trials on phage-mediated gut microbiota regulation, 14 of which were based on moderate to high-quality clinical evidence, indicating that research design in this field has advanced from the initial observational stage to the intervention verification stage.

**Conclusion:**

This research systematically sorts out the research progress of 20 years in the field of bacteriophage-mediated intestinal microbiota regulation through the method of literature metrology. The research clearly outlines the evolutionary trajectory of this field from basic description to mechanism exploration to clinical transformation. Future research should focus on the following directions: clarifying the molecular mechanism of the interaction of core diseases, establishing a standardized research framework, and carrying out large-scale multi-center clinical trials to promote the transformation of this field from basic research to clinical application.

## 1 Introduction

The human intestine is by no means a simple digestive tract, but an ecological universe woven by bacteria, archaea, fungi, viruses and protists (Lynch and Pedersen, 2016; Sommer and Bäckhed, 2013). In this microworld, the intestinal microbiota-often known as the “second genome” of the host-has long been regarded as a key hub for understanding metabolism, immunity and even neural function (Qin et al., 2010; Clemente et al., 2012). However, since 2005, with the gradual deepening of research in the field of intestinal microorganisms, scientists’ focus has gradually shifted from bacteria to their smaller but more destructive regulators-bacteriophages (De Sordi et al., 2019). These bacteria-infecting viruses represent the most abundant viral component of the gut microbiota, collectively forming the highly diverse and dynamic gut phageome (Minot et al., 2011). They are not only the “natural regulators” of microbiota, but also the “invisible engines” that drive gene flow and functional evolution in the community (Rohwer and Thurber, 2009). From inflammatory bowel disease to metabolic syndrome, there is more and more evidence that bacteriophage disorder may be a precursor to the occurrence and progression of the disease (Gogokhia et al., 2019). Precise regulation of this “viral community” may open up an unprecedented new way to reshape intestinal health (Federici et al., 2022).

Although this research field has experienced unprecedented rapid growth, this apparent prosperity masks a critical challenge: the burgeoning research outputs are dispersed across journals spanning diverse disciplines, including microbiology (Shkoporov and Hill, 2019), virology (Dion et al., 2020), clinical medicine (Rasmussen et al., 2020), and synthetic biology (Ando et al., 2015). This dispersion leads to a fragmentation of knowledge—with studies framed by distinct disciplinary paradigms, terminologies, and research foci, hindering cross-disciplinary integration, coherent dialogue, and the establishment of a unified theoretical framework for phage-mediated gut microbiota regulation. We must ask: What kind of development trajectory does this field follow? What research constitutes its basic theory? Where does the current cutting-edge research point? How can scholars in different fields carry out collaboration and dialogue? Although the traditional literature review can outline some pictures, it is difficult to fully present the panorama and dynamic trajectory of the field.

Here, bibliometrics provides a unique “bird’s-eye view.” Through the quantitative analysis of a large amount of academic literature, it objectively depicts the “scientific knowledge atlas” in this field, clearly revealing the migration of research hotspots, the inheritance vein of key publications, and the formation process of academic communities. This method has verified its value in many interdisciplinary fields, helping us identify the real “signal” from the “noise” of the data (Newman, 2004; Chen and Song, 2019; Newman, 2001).

Although previous studies have tried to apply this method to a wider range of bacteriophages (Liping et al., 2024; Dagli et al., 2024; Jiang et al., 2025), a systematic review of the core scientific problem “How bacteriophages regulate the intestinal microbiota” is still missing. This gap hinders our ability to determine the following issues: Is there a disconnect between basic mechanism exploration and clinical transformation? How can the innovation of technical methods concretely promote the transformation of the research paradigm? Do different research directions form synergistic forces, or do they operate in isolation?

Therefore, we aim to fill this gap. This study employs bibliometric methods to conduct a systematic “academic mapping” of the specific domain of “phage regulation of the gut microbiota.” We hope this work will not only trace the historical knowledge trajectory but also clearly identify current research frontiers and potential interdisciplinary convergence points, providing a valuable roadmap for future exploration of this complex and fascinating microscopic ecosystem.

## 2 Materials and methods

### 2.1 Data source and search strategies

**Figure 1:**
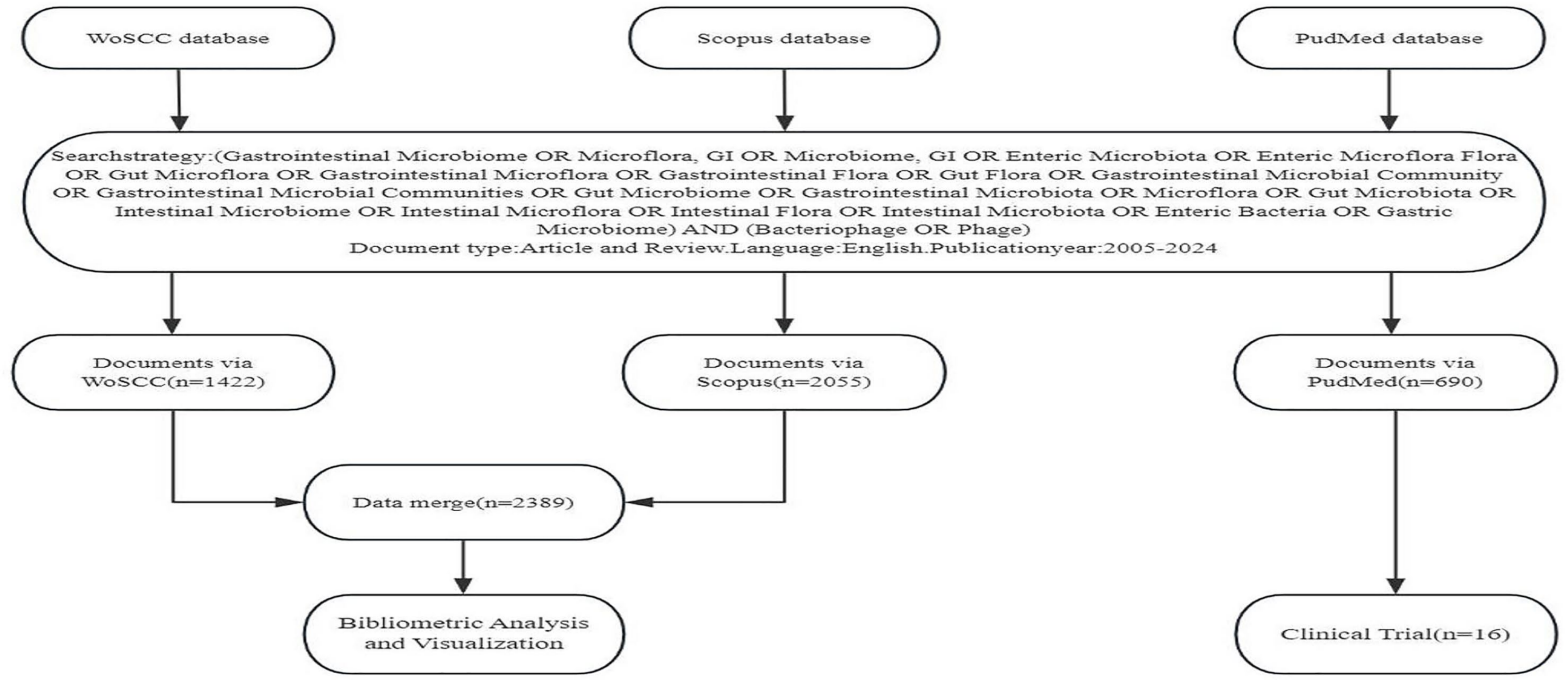
Flowchart of literature retrieval and data processing.

The data for this study were sourced from the Web of Science Core Collection (WoSCC), Scopus, and PubMed databases. Web of Science is the world’s leading citation index platform for natural sciences, social sciences, arts, and humanities, and also serves as an authoritative publisher-independent global citation database (Jiang et al., 2023). Scopus is an international abstract and citation database launched by Elsevier, covering multidisciplinary fields including natural sciences, medicine, and social sciences. The platform supports scientific research literature retrieval and index analysis through high-quality data and advanced analysis tools (Baas et al., 2020). PubMed is a free biomedical literature database maintained by the National Biotechnology Information Center of the United States, which is famous for its authority and open access (Canese and Weis, 2013). In order to improve the integrity, representativeness and accessibility of data, this study retrieves three major databases. The search time range is set from January 1, 2005, to December 31, 2024 to cover the literature published or indexed during this period. The specific retrieval strategy is shown in Figure 1.

### 2.2 Inclusion and exclusion criteria

This study adopted the following literature screening criteria: only original research papers and reviews that have undergone strict peer review and focus on bacteriophage regulation of intestinal microbiota were included to ensure the quality of literature and research relevance. Simultaneously, any literature meeting any of the following conditions was excluded: (1) Materials in the pre-publication stage; (2) non-systematic research materials such as conference abstracts or academic errata; (3) duplicated publications of the same research findings across different outlets; (4) articles not written in English. To ensure data authenticity, two qualified researchers independently screened and extracted key information from the final included literature before jointly analyzing the data. Discrepancies identified during the full-text review process were resolved through consultation.

### 2.3 Bibliometric and visualized analysis

To systematically analyze literature trends and research hotspots in the fields of phages and gut microbiota, this study employed multiple bibliometric tools for quantitative analysis: Microsoft Office Excel was used to record and organize annual publication trends and citation patterns, while the tracking and analysis of publication trends were conducted through manual verification combined with descriptive statistical summaries. While CiteSpace (Synnestvedt et al., 2005) performed citation analysis and visualization (scientific knowledge maps) to reveal the domain’s knowledge foundation, structural characteristics, and evolutionary trends. Through co-citation analysis and the use of the software’s significance detection function, we could identify high-impact publications (reference significance) and research topics (keyword significance). High reference significance represented the core research results of a specific period, while high keyword significance indicated the transformation of research frontiers and hot spots. This study used VOSviewer (van Eck and Waltman, 2010) for collaborative network analysis (revealing the cooperation mode between countries, authors and institutions), co-citation literature analysis of core journals, and co-presentation analysis of keywords (showing network relevance). Intensity of international collaboration, defined as the level of research engagement between different countries, was operationalized using total link strength (TLS). In this context, TLS quantifies the cumulative strength of collaborative publication links connecting a focal country to all other countries in the network, with higher values indicating more extensive international research partnerships. Visualization was realized for the time evolution and association intensity through time overlay layers and TLS. In addition, R-bibliometrix (R Core Team, 2013) was used to analyze the national collaborative network, the author’s time output mode and keyword trends. Through the collaborative application of these multi-dimensional methods, this research aimed to dynamically present the research hotspots, evolutionary trajectories and emerging trends in this field.

### 2.4 Research ethics

The data came from the public database and did not involve human or animal subjects. This literature measurement analysis did not need to be reviewed by the ethics committee.

### 2.5 Journal ranking and definition of top journals

Top journals mentioned in this study are defined as those journals that rank highly within their respective disciplines after sorting all journals in descending order based on the impact factor and citation score published by Clarivate. Consistent and explicit screening criteria were applied throughout all analyses to ensure a standardized and transparent research process, as well as reproducible and comparable results.

## 3 Results

### 3.1 Publication volume

**Figure 2:**
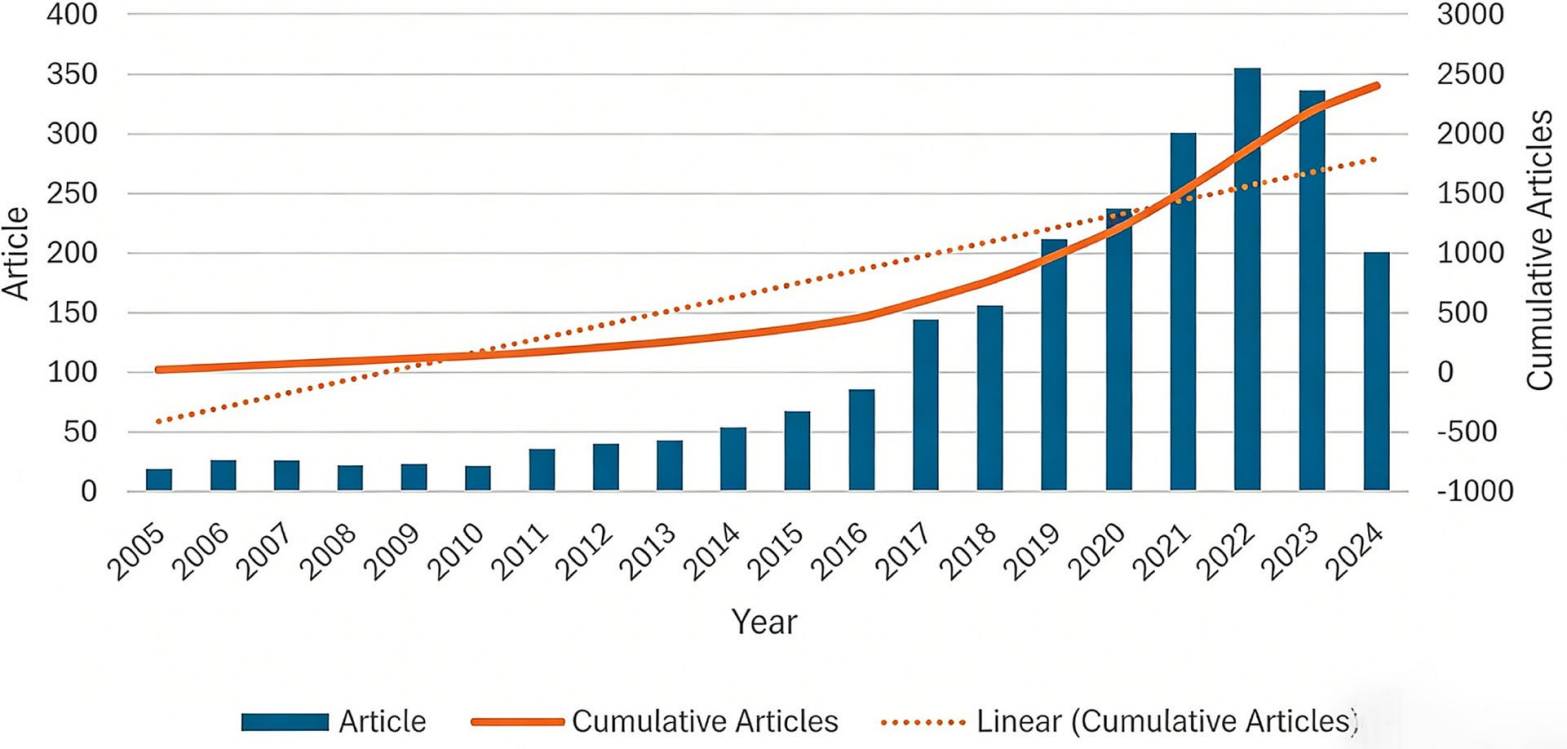
Annual publication volume and cumulative publication volume trend chart. The abscissa is the year (2005–2024), the left ordinate is the annual publication volume (Article), and the right ordinate is the cumulative publication volume (Cumarticle); the blue bar chart represents the annual publication volume, the orange solid line represents the cumulative publication volume, and the orange dotted line is the linear fitting line of the cumulative publication volume, which intuitively shows the annual fluctuation and long-term cumulative growth trend of publication volume in this field.

The research on bacteriophage regulation of intestinal microbiota has shown a significant growth trend in the past two decades. In terms of annual publication volume (Figure 2), the development of this field was relatively slow in the early stage (2005–2010), and the annual output was maintained at about 20–30 articles. Since 2011, the volume of publications has risen steadily, especially after entering the stage of rapid growth. The publication volume exceeded 140 articles in 2017, reaching a peak of 355 articles in 2022, reflecting the continuous increase in the attention of the academic community to this field. Although the number of publications decreased slightly in 2023 (336 articles), the overall volume is still at a high level. This shows that the bacteriophage-intestinal microbiota interaction mechanism and its potential application in disease treatment and regulation of the gut microbiota continue to attract the strong interest of researchers. The cumulative trend of publication shows a continuous growth trend, and the growth rate has accelerated significantly after 2016, indicating that relevant research has entered the outbreak period (Marchesi et al., 2016; Ogilvie and Jones, 2015).

### 3.2 Global research output distribution

From the perspective of national publication output, citation performance and international collaboration intensity (Table 1), the United States has an absolute advantage in this field, publishing a total of 695 papers (accounting for 29.09% of the total number of publications), with a total number of citations of 51,270, and a mean of 73.77 citations per paper. Its international cooperation performance is equally brilliant (total number of cooperation papers TLS = 409). China ranks second with 341 papers (14.27%), with a total of 13,429 citations and a mean of 39.38 citations per paper, but the intensity of international cooperation is relatively low (TLS = 123). Britain, Germany and France ranked third to fifth, respectively. The three countries have performed strongly in terms of total citations and the mean citations of each paper, and international cooperation is active. It is worth noting that although Ireland ranks eighth with only 85 papers, it has a mean of 89.44 citations per paper. Showing significant academic influence. Each paper in France, Italy and Spain has been cited more than 75 times, reflecting the generally high quality of research papers in this field in European countries.

**Table 1:** Top 10 productive countries regarding the research of phage regulation of gut microbiota.

Rank	Countries	Count (%)	Total citations	Average citations	TLS
1	USA	695 (29.09%)	51,270	73.77	409
2	China	341 (14.27%)	13,429	39.38	123
3	United Kingdom	157 (6.57%)	9,743	62.06	170
4	Germany	147 (6.15%)	9,534	64.86	173
5	France	139 (5.81%)	11,030	79.35	128
6	Australia	98 (4.10%)	6,630	67.65	111
7	Canada	91 (3.80%)	6,032	66.29	92
8	Ireland	85 (3.56%)	7,602	89.44	66
9	Italy	80 (3.35%)	6,042	75.53	91
10	Spain	79 (3.31%)	6,174	78.15	89

TLS, total link strength.

**Figure 3:**
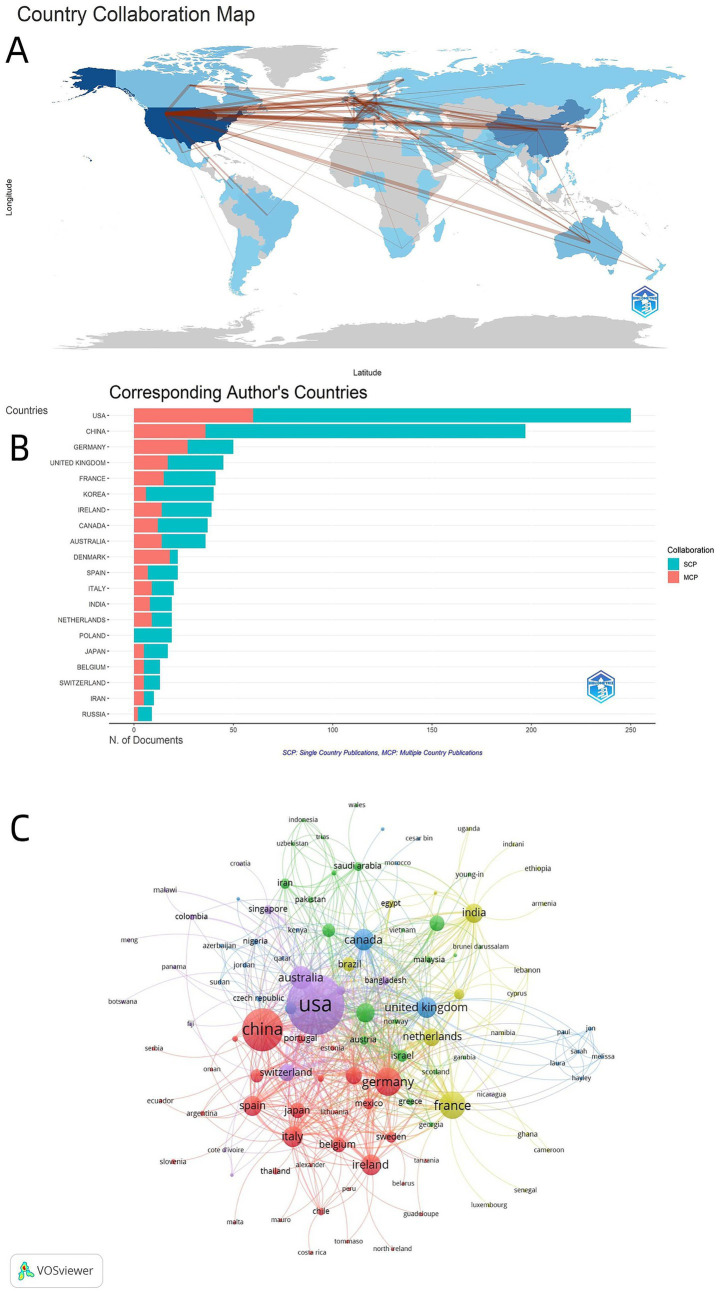
**(A)** International research collaboration map. The color depth of countries corresponds to the publication volume, and the line thickness reflects the collaboration frequency. **(B)** Corresponding author’s countries publication comparison. The abscissa is the publication volume, and the ordinate is the corresponding author’s country; cyan bars represent single country publications (SCP), and red bars represent multiple country publications (MCP). **(C)** Country collaboration network map. Node size corresponds to publication volume, color represents cluster groups, and line thickness reflects collaboration frequency (VOSviewer).

The “Global Collaborative Network Map” (Figure 3A) clearly shows the collaborative relationship between countries. The United States and China not only have the most collaborative nodes, but also have the widest distribution. These two countries naturally become the “core hubs” of the global collaborative network. The collaborative links radiating from these two core nodes show that international cooperation in this field is mainly concentrated in North America, Europe, East Asia and Australia. In the “Country Distribution of Corresponding Authors” chart (Figure 3B), the United States has the highest total number of papers published among all countries, of which SCP papers account for a significant proportion. China ranks second with the second highest total number of papers, and also shows a relatively high proportion of SCP papers. The number of papers published in Germany, Britain, France and other countries is in the middle, and the proportion of independent and cooperative papers is balanced. In general, the United States and China are the most active independent publishing countries in this field and actively participate in international cooperation. The number of papers published by other countries is relatively low, and the output of independent and cooperative papers is lower than that of the United States and China. “National Cooperation Network” diagram (Figure 3C) shows that the United States and China are the core hubs of international cooperation in this field and maintain intensive cooperative relations with many countries. The overall cooperation network covers many countries around the world, but it is mainly concentrated in major research countries in Europe, the Americas and Asia.

### 3.3 Research institution output and collaboration

**Figure 4:**
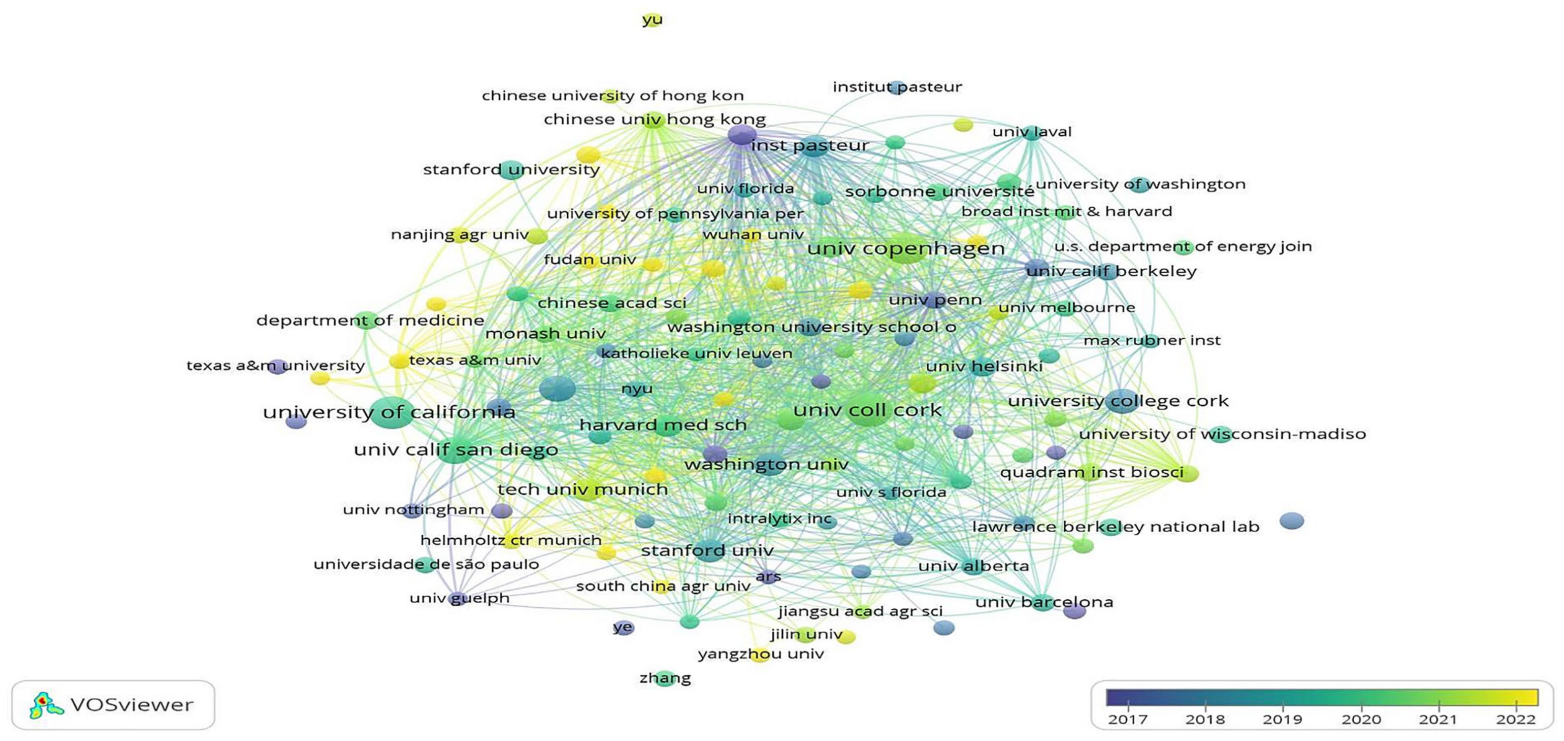
Evolution of the institutional co-citation collaboration network. Nodes represent research institutions, node size corresponds to publication volume, color corresponds to publication year, and line thickness reflects the collaboration frequency between institutions (VOSviewer).

From the perspective of institutional publication rankings, academic influence and collaborative network characteristics (Table 2), the University College Cork in Ireland topped the list with 58 papers published, while maintaining a high mean citation of 80.98 per paper (median: 41 citations per paper), fully demonstrating its excellent research results and academic influence in this field. American institutions dominate the list, with five institutions in the top 10. Among them, San Diego State University performed particularly well. Although it published only 19 papers, this institution achieved an outstanding mean citation of 188.26 per paper (median: 79 citations per paper). The research results of the University of Washington School of Medicine are equally brilliant as that of Stanford University, with the former achieving a mean citation of over 100 per paper (median: 54 citations per paper) and the latter a mean citation of over 100 per paper (median: 56 citations per paper). This shows that although the number of papers in some American universities may not be as high as that of top institutions, the quality and academic impact of their research results are of great value. In addition, the University of Copenhagen in Denmark, the Pasteur Institute in France and the Polish Academy of Sciences are also on the list, reflecting the active participation of European research institutions in this field. It is worth noting that the mean citation of each paper at the Technical University of Munich in Germany and the University of Barcelona in Spain is relatively low, which may mean that there is still room for improvement in the academic impact of their research results. The diagram of “Institutional Collaboration Network” (Figure 4) presents the core nodes of the network include the University of Copenhagen, the University of Cork, Stanford University and the University of California. These institutions show a dense collaborative network, highlighting their dual roles: they are not only the main contributors to scientific research output, but also the key hub of international cooperation in this field.

**Table 2:** Top 10 related institutions regarding the research of phage regulation of gut microbiota.

Rank	Institution	Country	Count (%)	Total citations	Average citations
1	University College Cork	Ireland	58 (2.42%)	4,697	80.98
2	University of California	USA	55 (2.30%)	3,992	72.58
3	University of Copenhagen	Denmark	45 (1.88%)	2,370	52.67
4	Stanford University	USA	28 (1.17%)	2,849	101.75
5	Washington University School of Medicine	USA	28 (1.17%)	3,673	131.18
6	Institut Pasteur	France	21 (0.88%)	1,786	85.05
7	Polish Academy of Sciences	Poland	21 (0.88%)	1,599	76.14
8	Technical University of Munich	Germany	20 (0.84%)	688	34.40
9	San Diego State University	USA	19 (0.80%)	3,577	188.26
10	University of Barcelona	Spain	18 (0.75%)	573	31.83

### 3.4 The most influential authors and author collaborative network

Ireland-based institutions constitute the core group of high output in this field-Colin Hill tops the list with 42 papers and 3,652 total citations. Andrey N. Shkoporov and Paul Ross from the same Ireland-based institution are also in the top four. Their papers are not only published centrally, but the mean number of citations also remains high, reflecting the continuous investment and academic leadership of the Irish team in this field. Notably, Matthew B. Sullivan, a prominent scholar in the field with a career trajectory from MIT to the University of Arizona and currently affiliated with The Ohio State University (USA), despite publishing only 13 papers, he ranks among the top 10 most influential authors with a mean of 209.8 citations per paper (median: 90 citations per paper), highlighting the strong academic impact of his research outcomes and making him a typical example of “elite breakthrough” scholars in this field. Core authors from other countries exhibit the characteristics of “professional focus,” with representative scholars having established certain academic influence in this field (Table 3).

**Table 3:** Top 10 authors related to phage regulation of gut microbiota.

Rank	Author	Country	Documents	Citations	Average article citations	TLS
1	Colin Hill	Ireland	42	3,652	86.95	2,594
2	Andrey N. Shkoporov	Ireland	27	2,748	101.77	1,974
3	Paul Ross	Ireland	24	1,171	48.79	1,443
4	Lorraine A. Draper	Ireland	20	1,435	71.75	1,448
5	Jillian F. Banfield	USA	17	1,310	77.05	140
6	Siew Chien Ng	China	15	1,542	102.80	583
7	Dennis Sandris Nielsen	Denmark	15	657	43.80	544
8	Laurent Debarbieux	France	14	844	60.29	546
9	Matthew B. Sullivan	USA	13	2,727	209.80	371
10	Andrzej Gorski	Poland	13	531	40.85	355

**Figure 5:**
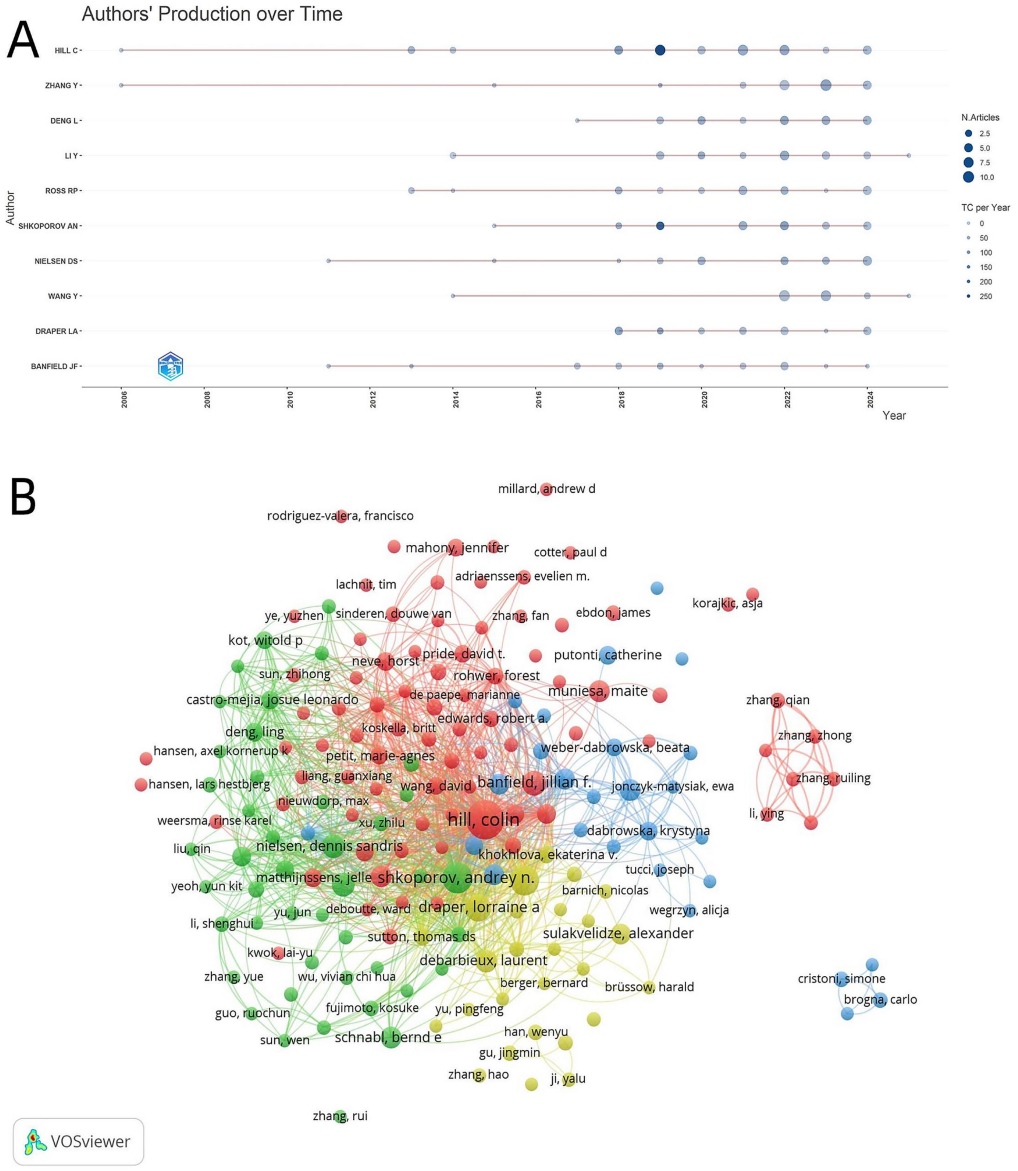
**(A)** Evolution of author production over time. The abscissa is the year, and the ordinate is core authors; the color depth of dots corresponds to the number of articles, and the size of dots corresponds to the annual publication volume) (N. Articles = Number of Articles, TC per Year = Total Citations per Year). **(B)** Author collaboration network map. Nodes represent authors, node size corresponds to publication volume, color corresponds to cluster groups, and line thickness reflects collaboration frequency between authors (VOSviewer).

Further analysis combined with the annual output trend (Figure 5A) and the author collaboration network (Figure 5B) shows that Colin Hill and Andrey N. Shkoporov act as “hub nodes” in the collaboration network. Their cooperative ties are extremely strong: they have not only formed stable research clusters with local teams but also established frequent cooperative contacts with scholars from multiple countries, effectively weaving a transnational collaborative network centered on core scholars. Meanwhile, the steady growth in annual publication volumes and citation counts of core scholars such as Colin Hill and Andrey N. Shkoporov demonstrates that this group has consistently deepened their engagement in this field, with continuous accumulation of research momentum and academic influence (Figure 5A).

### 3.5 The most influential journal

Frontiers in Microbiology has become the core publication platform in this field with a publication volume of 129 papers. However, its mean citations per paper are only 10.79 (median: 6 citations per paper), reflecting the characteristic of “high publication volume and broad coverage.” In contrast, Q1 journals such as Microbiome, Nature Communications, and Gut Microbes, although with relatively limited publication volumes, have mean citations per paper exceeding 37. Specifically, Nature Communications has a mean citation of 70.93 per paper (median: 40 citations per paper), and its 2025 impact factor falls within the high range of 11–15.7. These journals highlight themselves as the primary carriers of high-impact research outcomes in this field, representing the cutting-edge level of the research (Table 4).

**Table 4:** Top 10 productive journals related to phage regulation of gut microbiota.

Rank	Journal	Count	Total citations	Average citations	Impact factor (2025)	JCR
1	Frontiers in Microbiology	129	4,392	10.79	4.5	Q2
2	Microbiome	58	4,082	70.38	12.7	Q1
3	Gut Microbes	48	1,813	37.77	11	Q1
4	Nature Communications	43	3,050	70.93	15.7	Q1
5	mSystems	42	951	22.64	4.6	Q1
6	Frontiers in Cellular and Infection Microbiology	41	1,481	36.12	4.8	Q1
7	Microorganisms	40	710	17.75	4.2	Q2
8	Applied and Environmental Microbiology	38	992	26.11	3.7	Q2
9	Viruses	37	1,352	36.54	3.5	Q2
10	Scientific Reports	36	1,151	31.97	3.9	Q1

JCR, journal citation reports.

**Figure 6:**
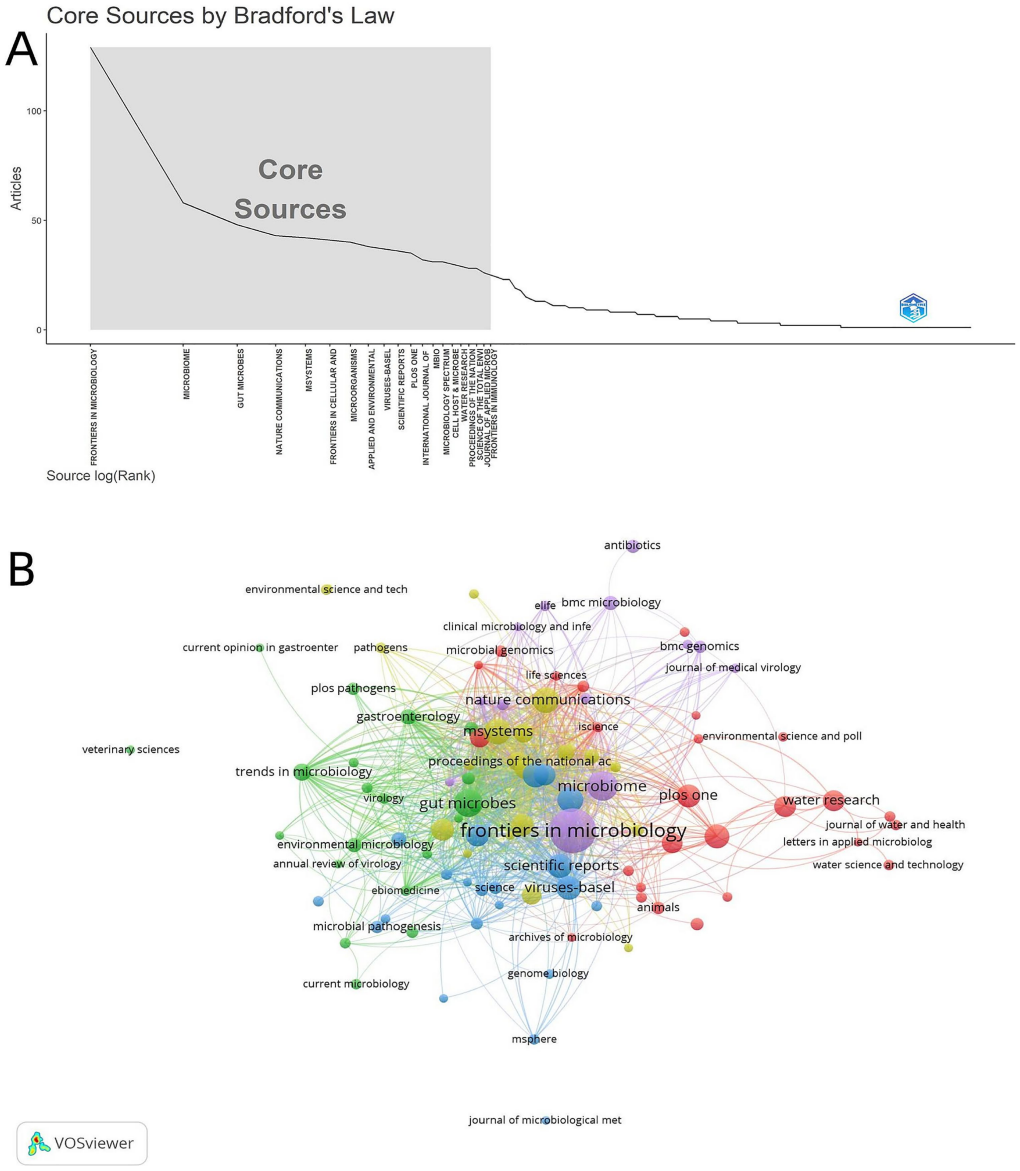
**(A)** Core sources identified by Bradford’s law. The abscissa is the source log rank, and the ordinate is the source publication volume; the curve fitting result divides the “Core Sources” region. **(B)** Journal collaboration network map. Nodes represent literature sources, node size corresponds to publication volume, color corresponds to cluster groups, and line thickness reflects the co-occurrence frequency between different journals (i.e., the number of times two journals are simultaneously associated with the same research article) (VOSviewer).

Core journal distribution map constructed based on Bradford’s law (Figure 6A): The steep attenuation curve presented in the “core source” area clearly reveals the characteristics of literature distribution in this field-top journals such as Frontiers in Microbiology and Microbiome maintain high output. With the decline in the ranking of journals, the number of papers decreased sharply. This intuitive trend is highly consistent with the “core-dispersion” law of Bradford’s law, which directly shows that the vast majority of literature in this field is highly concentrated in a few core journals. These core-region journals also serve as the primary carriers for the dissemination and exchange of academic achievements in this field.

Frontiers in Microbiology and Microbiome form a network with journals like Gut Microbes and Viruses-Basel through dense connecting lines, and the network is intertwined around thematic keywords including “microbiome,” “gut microbiota,” and “virology” (Figure 6B). This characteristic not only indicates that the core journals in this field have formed a stable cluster, but also reflects that research on phage-mediated gut microbiota regulation is not isolated. Instead, it has in-depth thematic intersections with adjacent fields such as microbiology and virology. The linkage between journals also provides support for the circulation of cross-disciplinary academic resources.

### 3.6 Keyword analysis

**Figure 7:**
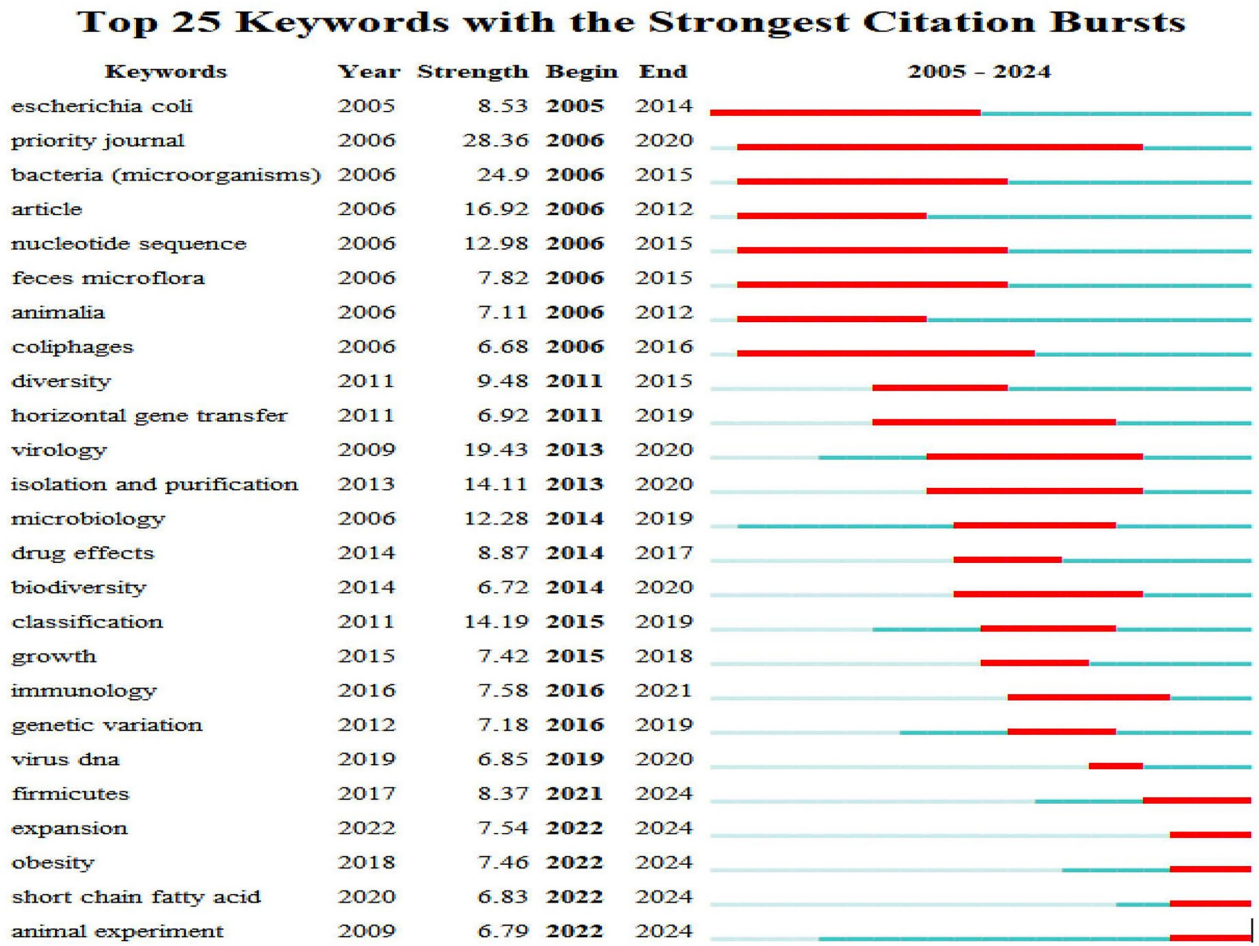
Top 25 keywords with the strongest citation bursts. The abscissa is the year (2005–2024), and the ordinate is the top 25 keywords by citation burst strength; red line segments represent the active burst period of keywords, with their time span corresponding to the start and end years of the burst, and burst strength values are directly labeled in the table (CiteSpace).

From the temporal distribution of high-burst keywords (Figure 7), the research focus from 2005 to 2010 centered on basic directions such as *Escherichia coli*, “nucleotide sequence,” and “bacteria (microorganisms).” Studies during this phase mainly involved the classification and identification of bacteriophages and gut microbes, as well as gene sequence analysis, marking the technical foundation-laying stage of the field’s development. Between 2011 and 2018, the keywords “diversity” and “horizontal gene transfer” entered the high-frequency outbreak period. It is worth noting that the outbreak period of “horizontal gene transfer” (2011–2019) is highly consistent with the surge period of bacteriophage-mediated gene transfer research in the intestinal microbiota, reflecting the transformation of this field from basic description to mechanism exploration (Mukhopadhya et al., 2019). At the same time, “virology” entered a high outbreak period in 2013 and continued to be active until 2020, further expanding the dimension of mechanism research at this stage. After 2021, function-oriented keywords such as “thick-walled bacteria,” “obesity” and “short-chain fatty acids” have become new hot spots: “short-chain fatty acids” have been highly concerned since 2022 (to 2024), which is directly related to the regulation between intestinal microbial metabolites and host health mechanism; The outbreak period of “thick-walled bacteria” and “obesity” both span 2022–2024, pointing to the research direction of the relationship between the structure of intestinal microbiota and metabolic diseases. In addition, the long-term emergence of “priority journals” from 2006 to 2020 reflects the continuous attention to high-impact achievements in this field and the formation of academic consensus.

**Figure 8:**
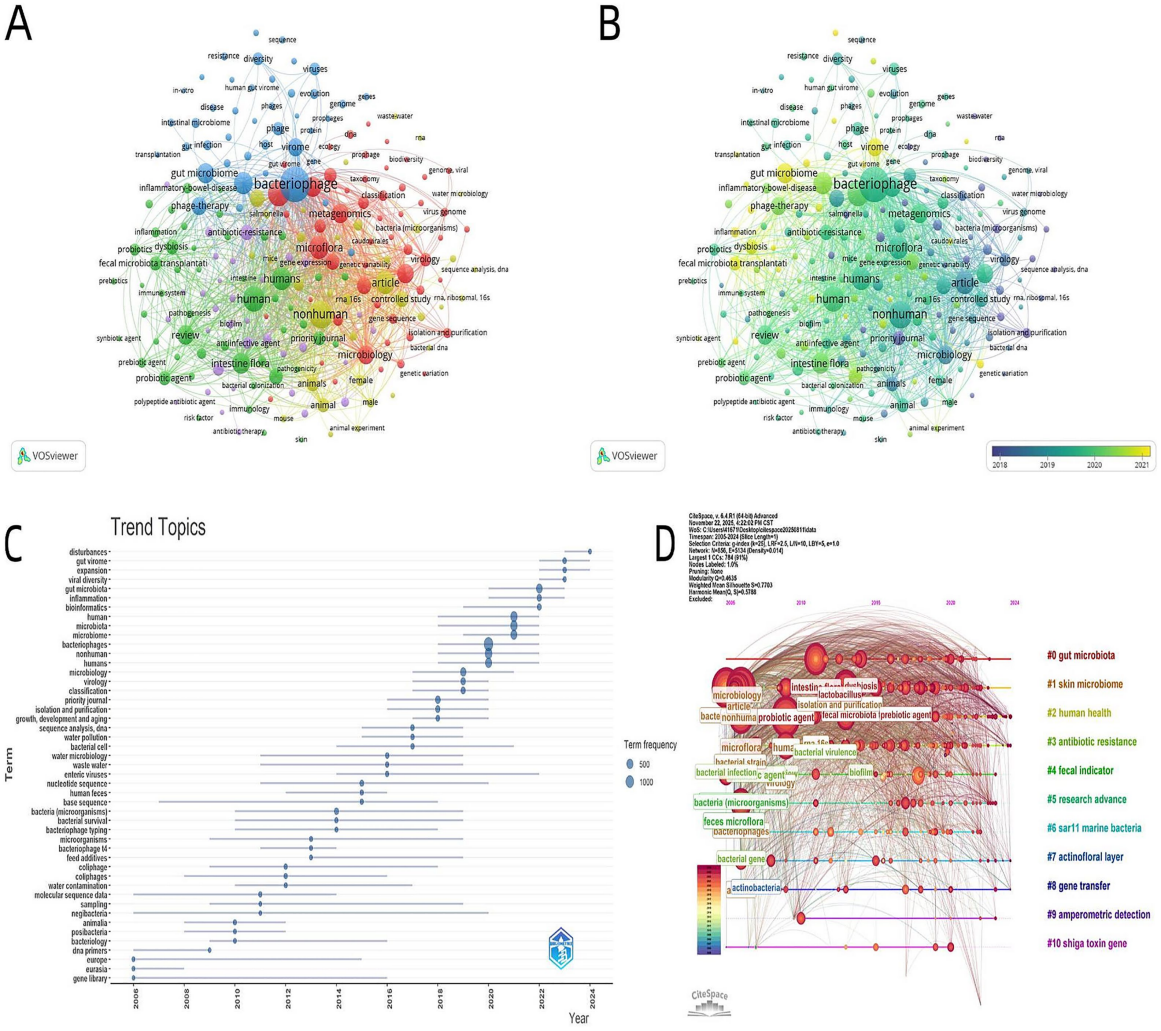
**(A)** Keyword co-occurrence network map. Nodes represent keywords, node size corresponds to occurrence frequency, color corresponds to cluster groups, and line thickness reflects keyword co-occurrence frequency (VOSviewer). **(B)** Evolution of the keyword co-occurrence network map. Nodes represent keywords, node size corresponds to occurrence frequency, color corresponds to the year of keyword occurrence, and line thickness reflects keyword co-occurrence frequency (VOSviewer). **(C)** Temporal distribution and evolution of research topics. The abscissa is the year, and the ordinate is hot keywords; the size of dots corresponds to keyword occurrence frequency, and the horizontal line above each dot represents the active time span of the keyword. **(D)** Time-zone evolution map of research themes. Nodes represent subject keywords, where the size of the node corresponds to the word frequency, the color corresponds to the time interval when the subject appears, and different cluster groups (#0–#10) are marked with different colors (CiteSpace).

The keyword co-present network diagram (Figures 8A,B) further reveals the radiation range of the core theme. Bacteriophage and gut microbiota form dual core nodes, closely connected with associated keywords such as phage therapy, antibiotic resistance, and metagenomics. Among these, metagenomics has the highest connection density, confirming the core supporting role of this technology in analyzing bacteriophage-gut microbiota interactions (Figure 8A). The 2018–2021 stratified map (Figure 8B) shows that the radiation scope of the core nodes bacteriophage and gut microbiota has expanded significantly, adding direct connections with dysbiosis and human in 2019–2020: the integration of dysbiosis reflects the shift from “describing microbiota characteristics” to “regulating microbiota homeostasis,” while the strengthening of the human node marks the focus of research scenarios shifting from model organisms to human applications, reflecting the pragmatic development trend of the field.

The timeline feature of the trend topic map (Figure 8C) intuitively presents the dynamic ebb and flow of keyword popularity. Before 2010, keywords such as gene library and Europe had relatively high occurrence frequencies: the former corresponds to the technical needs of early gene sequencing, while the latter reflects the regional concentration characteristic of research outputs in this phase. After 2020, the frequency curves of gut virome disturbance, viral diversity, and bioinformatics showed a steep upward trend: gut virome disturbance has gained attention mainly because researchers recognize that bacteriophages are key components of the gut virome and aim to clarify how they regulate microbiota structure; the rising popularity of bioinformatics is driven by the growing volume of metagenomic and metaviromic data, which requires this technology for analysis and processing—this also reflects that research questions and technical methods are mutually reinforcing.

The keyword timeline map (Figure 8D), through the coupling of node connection strength and temporal dimension, clearly presents the deepening process of the field’s research mainline. From the map’s characteristics, gut microbiota (as a red-labeled core node) runs through the entire research period, and the evolution of its associated keywords directly reflects the shift in research focus: before 2010, the associated terms of this node were concentrated in basic taxonomy categories such as bacterial strain and microflora; combined with relevant data, it can be concluded that research in this phase focused on the composition description and classification identification of gut microbiota (Human Microbiome Project Consortium, 2012). After 2020, the node connection density between gut microbiota and probiotics, fecal microbiota transplantation, and antibiotic resistance increased significantly—this characteristic corresponds to the substantial expansion of research directions: the triangular connection structure formed by fecal microbiota transplantation, bacteriophage, and gut microbiota confirms, from a bibliometric perspective, that the field has initiated exploration of bacteriophage-mediated regulation of post-transplant microbiota homeostasis (Zuo and Ng, 2018; Zhang et al., 2019); the direct connection between antibiotic resistance and bacteriophage reflects that the research demand for bacteriophages as an intervention for drug-resistant bacteria has formed an academic consensus (Strathdee et al., 2023). Notably, since 2022, the bacteriophage node has moved closer to the mainline of “gut microbiota-host health,” and its indirect connection strength with obesity and short-chain fatty acid has increased. This map characteristic marks that the research paradigm has officially transitioned from “bacteriophage-microbiota” dual interaction analysis to a “bacteriophage-microbiota-host health” tripartite association research system (Federici et al., 2021)^.^

### 3.7 Characteristics of core co-cited literatures

From the top 15 co-cited literatures it can be seen that these are the most frequently co-cited achievements in the field (Table 5): Disease-specific alterations in the enteric virome in inflammatory bowel disease, published by Jason M. Norman in Cell in 2015 (Norman et al., 2015), ranks first with a total of 215 citations, focusing on the association between the gut virome and diseases. The 2010 fecal virome study by Alejandro Reyes published in Nature (Reyes et al., 2010), and the 2011 research on individual differences in the gut virome by Samuel Minot constitute the core literatures of the early “characterization of gut virome features” in the field (Minot et al., 2011). Meanwhile, the 2013 paper by Jeremy J. Barr proposing that “bacteriophages adhere to mucous membranes to provide non-host-derived immunity” serves as a key theoretical foundation for the functional regulation of gut microbiota by bacteriophages (Barr et al., 2013).

**Table 5:** Top 15 co-cited references related to phage regulation of gut microbiota.

Rank	Title	Year	Total citations	First author	Journal
1	Disease-specific alterations in the enteric virome in inflammatory bowel disease	2015	215	Jason M. Norman	Cell
2	Viruses in the fecal microbiota of monozygotic twins and their mothers	2010	214	Alejandro Reyes	Nature
3	The human gut virome: inter-individual variation and dynamic response to diet	2011	188	Samuel Minot	Genome Research
4	Bacteriophage adhering to mucus provide a non-host-derived immunity	2013	156	Jeremy J. Barr	Proceedings of the National Academy of Sciences of the United States of America
5	The human gut virome is highly diverse, stable, and individual specific	2019	146	Andrey N. Shkoporov	Cell Host & Microbe
6	Healthy human gut phageome	2016	132	Pilar Manrique	Proceedings of the National Academy of Sciences of the United States of America
7	Bacteriophages of the human gut: the “known unknown” of the microbiome	2019	131	Andrey N. Shkoporov	Cell Host & Microbe
8	Rapid evolution of the human gut virome	2013	126	Samuel Minot	Proceedings of the National Academy of Sciences of the United States of America
9	Dynamic modulation of the gut microbiota and metabolome by bacteriophages in a mouse model	2019	122	Bryan B. Hsu	Cell Host & Microbe
10	A highly abundant bacteriophage discovered in the unknown sequences of human fecal metagenomes	2014	121	Bas E. Dutilh	Nature Communications
11	Expansion of bacteriophages is linked to aggravated intestinal inflammation and colitis	2019	116	Lasha Gogokhia	Cell Host & Microbe
12	Early life dynamics of the human gut virome and bacterial microbiome in infants	2015	114	Efrem S. Lim	Nature Medicine
13	Fast gapped-read alignment with Bowtie 2	2012	112	Ben Langmead	Nature Methods
14	The gut virome database reveals age-dependent patterns of virome diversity in the human gut	2020	108	Ann C. Gregory	Cell Host & Microbe
15	Gnotobiotic mouse model of phage-bacterial host dynamics in the human gut	2013	100	Alejandro Reyes	Proceedings of the National Academy of Sciences of the United States of America

**Figure 9:**
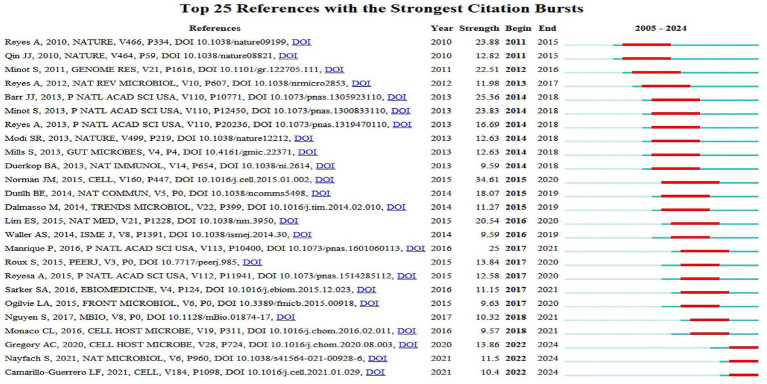
Top 25 references with the strongest citation bursts. The abscissa is the year (2005–2024), and the ordinate is the top 25 references by citation burst strength; red line segments represent the active burst period of the reference, with their time span corresponding to the start and end years of the burst, and burst strength values are directly labeled in the table (CiteSpace).

After 2019, multiple papers by Andrey N. Shkoporov on gut virome stability and bacteriophage-microbiota interactions entered the top 15, reflecting the deepening of research toward “mechanisms of bacteriophage-microbiota interactions” during this phase (Shkoporov and Hill, 2019; Shkoporov et al., 2019). The burst pattern of literatures is evident (Figure 9): 2010–2013 was the burst phase of early core literatures, with the burst strengths of Alejandro Reyes and Samuel Minot exceeding 20 Minot et al. (2011) and Reyes et al. (2010), and persisting until 2015–2018. After 2015, Jason M. Norman’s paper became a new focus with a burst strength of 34.61 (Norman et al., 2015). From 2020 to 2021, literatures on gut virome databases and phageome analysis by Nayfach et al. (2021) began to burst, corresponding to the development of technical tools.

**Figure 10:**
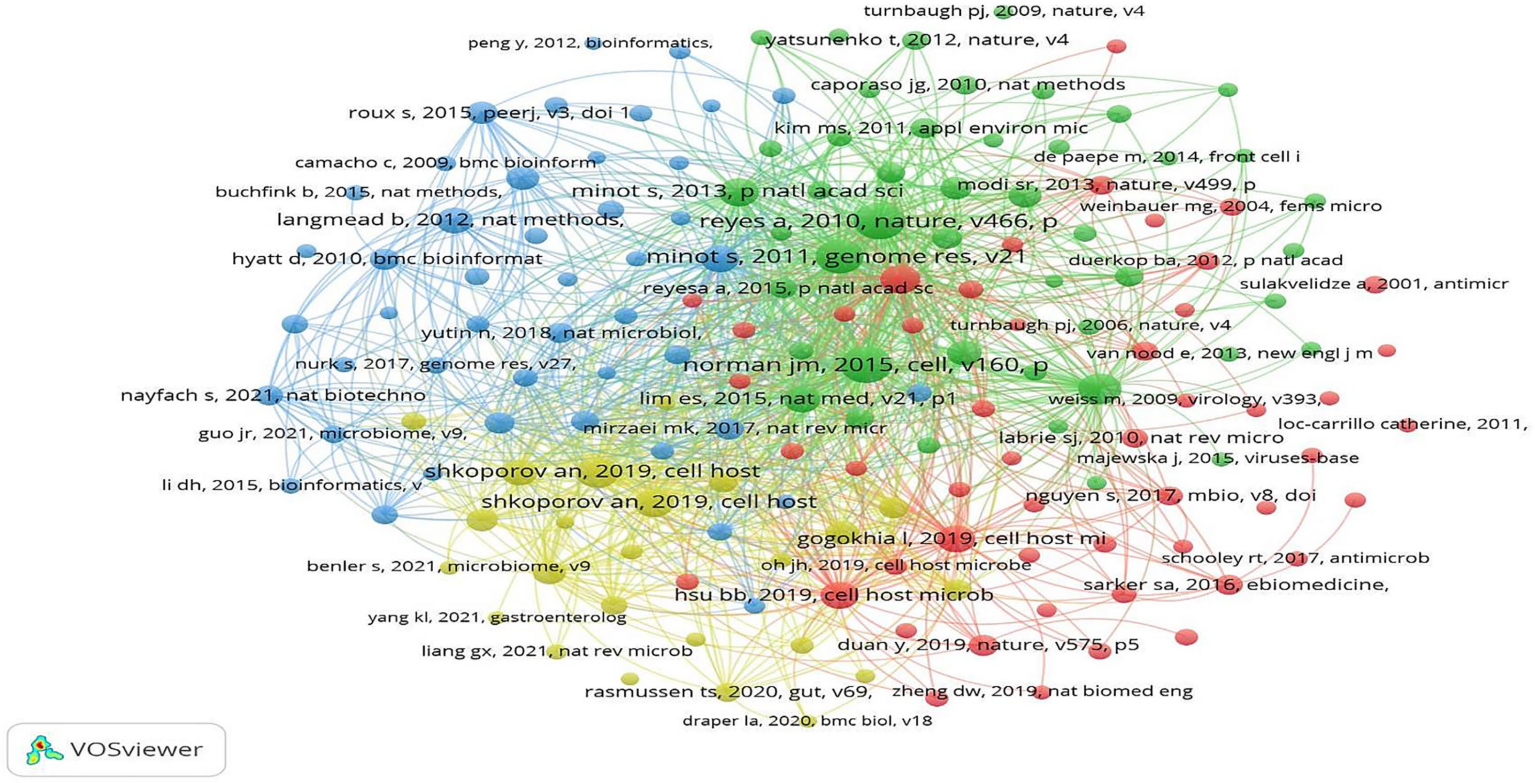
Co-cited references network map. Nodes represent individual high-impact references, node size corresponds to their citation frequency or co-occurrence count, color corresponds to cluster groups, and line thickness reflects co-occurrence frequency.

From the perspective of the literature co-occurrence network map (Figure 10), nodes are clustered based on co-citation relationships: the blue cluster mainly includes literatures on bioinformatics tools, the green cluster corresponds to early studies on gut virome characteristics, the red cluster focuses on research on the association between bacteriophage-microbiota interactions and diseases, and the yellow cluster concentrates recent achievements by scholars such as Andrey N. Shkoporov after 2019. The high connection density between different clusters indicates that the core literatures in the field have not only formed their respective research branches but also maintained close academic correlations.

### 3.8 Analysis of relevant clinical trials in the PubMed database

A literature integration analysis covering the period 2008–2024 identified 16 English-language clinical trials and animal study reports that met the inclusion criteria, including 8 human clinical trials (Atterbury et al., 2011; Sarker et al., 2017; Zuppi et al., 2024; Wortelboer et al., 2023; Ma et al., 2023; Furber et al., 2022; Leo et al., 2021; Grubb et al., 2020), 2 animal experiments (Kang et al., 2017; Majzoub et al., 2024), and 6 clinical observational studies (Inoue et al., 2008; Manrique et al., 2021; Trotter et al., 2020; Febvre et al., 2019; Zheng et al., 2019; Gindin et al., 2019). From a temporal distribution perspective, 8 articles were included between 2005 and 2022, and this number increased to 8 between 2022 and 2024. This trend reflects the rapid evolution of the research paradigm in this field: with the popularization of macrogenome sequencing and multi-omics technology, scholars have shifted from the early study of the single pair of bacteriophages to the systematic exploration of the core role of intestinal bacteriophages in microbial homeosis, disease pathogenesis and intervention response (Garmaeva et al., 2019).

In terms of the distribution of journals and institutions, 16 papers have been published in 12 journals, of which 5 have been published in 2 core journals. These core journals include Microbiology (3 articles) and Nature Communications (2 articles), both of which are top journals in the fields of microbiology and gastroenterology, with stable influence. All published journals belong to the JCR Q1 level, covering microbiology (Zuppi et al., 2024; Wortelboer et al., 2023; Ma et al., 2023; Inoue et al., 2008; Trotter et al., 2020; Gindin et al., 2019), gastroenterology (Atterbury et al., 2011; Zuppi et al., 2024; Furber et al., 2022; Trotter et al., 2020), nutrition (Sarker et al., 2017; Wortelboer et al., 2023; Ma et al., 2023; Grubb et al., 2020), sports medicine (Leo et al., 2021) and infectious diseases (Kang et al., 2017; Majzoub et al., 2024; Inoue et al., 2008) and other fields fully reflect the interdisciplinary nature and international recognition of the research.

The study involves 25 research institutions around the world, mainly in Australia, the Netherlands, China, the United Kingdom and other regions. Among them, the University of New South Wales in Australia (Zuppi et al., 2024; Trotter et al., 2020) and Amsterdam UMC in the Netherlands (Sarker et al., 2017; Ma et al., 2023) each contributed 2 relevant articles, while the remaining institutions were independent research units. In addition, there were 6 cross-border collaborative studies, reflecting the international collaborative characteristics and global research network of the field.

Randomized controlled trials (RCTs) dominated the study design types, with a total of 8 articles, including 6 double-blind RCTs (Atterbury et al., 2011; Zuppi et al., 2024; Wortelboer et al., 2023; Ma et al., 2023; Furber et al., 2022; Leo et al., 2021) and 2 single-blind RCTs (Sarker et al., 2017; Grubb et al., 2020); 3 clinical observational studies (Manrique et al., 2021; Febvre et al., 2019; Gindin et al., 2019); 2 animal experiments (Kang et al., 2017; Majzoub et al., 2024); and 3 multicenter trials (Atterbury et al., 2011; Zuppi et al., 2024; Leo et al., 2021). Fourteen of the sixteen studies provided moderate-to-high-level clinical evidence (defined as mid-to-high level study designs involving randomized controlled trials and cohort studies per the GRADE criteria for clinical evidence grading), but only 3 were large-sample studies (Sarker et al., 2017; Zuppi et al., 2024; Wortelboer et al., 2023). This indicates that the field has advanced from preliminary observation to the intervention verification stage, but the sample size still needs to be expanded.

The disease and health themes formed a dual-track pattern of “disease treatment + health optimization”: inflammatory bowel disease (Atterbury et al., 2011; Zuppi et al., 2024; Inoue et al., 2008; Trotter et al., 2020) and metabolism-related diseases (Wortelboer et al., 2023; Ma et al., 2023; Leo et al., 2021; Grubb et al., 2020) accounted for the highest proportion (4 articles each); 3 studies focused on gut microbial homeostasis in healthy populations (Manrique et al., 2021; Febvre et al., 2019; Zheng et al., 2019); 2 targeted intervention observations in high-risk groups (Wortelboer et al., 2023; Ma et al., 2023); 1 on chronic constipation (Furber et al., 2022); and 1 on avian Salmonella infection (Majzoub et al., 2024).

In terms of outcome measures, a complete three-tier observation system of “microbial structure-functional metabolism-clinical outcomes” was formed: all 16 articles detected phageome (vOTU abundance, diversity, composition) and bacterial community structure indicators; 12 articles (Atterbury et al., 2011; Sarker et al., 2017; Zuppi et al., 2024; Wortelboer et al., 2023; Ma et al., 2023; Furber et al., 2022; Leo et al., 2021; Grubb et al., 2020; Kang et al., 2017; Inoue et al., 2008; Manrique et al., 2021; Trotter et al., 2020) simultaneously measured functionally relevant indicators, including short-chain fatty acids (SCFAs), B-vitamin complexes, inflammatory factors (CRP, cytokines), and metabolites (blood glucose, blood lipids, amino acid derivatives); 10 articles (Atterbury et al., 2011; Sarker et al., 2017; Zuppi et al., 2024; Wortelboer et al., 2023; Ma et al., 2023; Furber et al., 2022; Leo et al., 2021; Grubb et al., 2020; Inoue et al., 2008; Trotter et al., 2020) included clinical outcome indicators (e.g., remission rate of ulcerative colitis, defecation frequency, endurance time, blood glucose fluctuations), realizing full-chain verification from “structural changes” to “functional impacts” and further to “clinical benefits.”

Regarding technical methods, metagenomic sequencing became the absolute mainstream technology, with 13 articles (Atterbury et al., 2011; Sarker et al., 2017; Zuppi et al., 2024; Wortelboer et al., 2023; Ma et al., 2023; Furber et al., 2022; Leo et al., 2021; Inoue et al., 2008; Manrique et al., 2021; Trotter et al., 2020; Febvre et al., 2019; Zheng et al., 2019; Gindin et al., 2019) adopting it to analyze phageome and microbial community structure; 2 articles (Kang et al., 2017; Majzoub et al., 2024) combined 16S rRNA sequencing with bacteriophage isolation and culture; and 1 article (Grubb et al., 2020) used targeted sequencing + metabolomics (LC-MS/GC-MS). Bioinformatics tools were concentrated on MEGAHIT (assembly), CheckV (viral genome assessment), VirSorter2 (bacteriophage identification), etc., indicating mature and centralized application of technologies, with an obvious trend of integrating genomics and metabolomics multi-omics approaches.

In summary, research on bacteriophage-mediated gut microbiota regulation has continued to gain momentum in recent years. The research paradigm has shifted from single observation to intervention verification, technical methods from traditional culture to multi-omics integration, and the observation system from structural description to full-chain verification, with 14 of the 16 included clinical trials providing moderate-to-high-level clinical evidence. However, there are still shortcomings such as insufficient large-sample RCTs, scarce research on certain diseases, and inadequate exploration of bacteriophage-bacteria interaction mechanisms. Future research should further strengthen multi-center, large-sample collaboration, focus on core diseases to conduct mechanism-oriented intervention studies, and promote the leap of this field from basic research to clinical translation and application.

## 4 Discussion

In this study, bibliometric methods were employed to systematically integrate analytical tools such as CiteSpace, VOSviewer, and R-bibliometrix, thereby conducting a multi-dimensional analysis of the evolutionary trends in the field of bacteriophage-mediated gut microbiota regulation from 2005 to 2024. Hereinafter, based on the bibliometric results and combined with the current research status of the field, an in-depth discussion will be carried out from two aspects: a summary of research characteristics and future development directions.

### 4.1 Systematic summary of research characteristics

#### 4.1.1 The national layer is differentiated into momentum, and the institutional path proves the effectiveness

The significant geographical differentiation pattern can be seen from the output at the national level compared with the outcome of influence. The United States has established a comprehensive dominant model of “high output-broad cooperation-strong impact.” Its 695 articles, 51,270 total citations and the highest intensity of international cooperation (TLS = 409) have jointly consolidated its position as the core hub of the global academic network. The success of this model comes from its continuous investment, open collaboration and leadership at the forefront of clinical transformation. In contrast, China shows the stage characteristics of “high output-to be improved-limited cooperation.” Although the number of articles published (341 articles) ranks second, 53.4% of the citation count of the United States, and the intensity of international cooperation is relatively limited. This outcome suggests that China has achieved catch-up in this field, but it is still a key challenge in the future in terms of enhancing the international influence of achievements and deep integration into global innovation networks. Europe represents the differentiated success path of “boutique-high quality.” Represented by Ireland, France and Italy, although the total output is not superior, it has gained higher mean influence with the depth and originality of research. This comparison reveals the three different national competition strategies in the field and their current results.

This model has been further confirmed at the institutional level. The University of Cork in Ireland (high output, high citation) and San Diego State University (low output, extremely high citation) represent the two successful institutional development paths of “continuous cultivation” and “precise breakthrough” respectively. The success of the former lies in building a stable core team and an extensive cooperation network; the latter proves that excellent innovation in key directions can bring high academic returns. The analysis of the institutional cooperation network diagram further shows that becoming the core node of the cooperative network (such as the University of Cork, the University of Copenhagen, Stanford University) is closely related to the improvement of institutional influence. “Open collaboration” is a significant success factor in amplifying research effectiveness.

#### 4.1.2 The core aggregation characteristics of the cooperative network and its efficiency differences

The analysis of the author’s cooperation network not only reveals the existence of core scholars, but also maps two highly influential differentiated cooperation models and their outcomes. With Colin Hill and Andrey N. Shkoporov Represented by the group of scholars, it shows the successful model of “core radiation type.” As the super hub of the network, they not only maintain in-depth collaboration within the team, but also establish extensive contacts with scholars from the United States, China, Britain and other countries, building an intensive cooperation network across the Atlantic. The successful outcome of this model is the combination of high productivity and high sustainable influence. The key is the efficient integration of internal and external resources through core nodes.

In contrast, American scholars Matthew B. Sullivan represented by the “elite alliance” model. It is characterized by the fact that the scope of cooperation may be relatively concentrated, but it has also achieved great academic success through small output but subversive high-impact results. These two models show that in this field, whether it is by building a broad collaborative ecology or focusing on building an elite breakthrough team, it can lead to the peak of academic influence. However, network analysis also suggests that the visibility and influence of teams whose research results are on the edge of cooperative networks or only cooperate internally are generally limited. Therefore, actively embedding or building a core cooperation network has become a dominant and successful strategy to improve the visibility and influence of research.

#### 4.1.3 Functional differentiation and influence hierarchy of journals: the “dual-track system” of achievement dissemination

The distribution characteristics of core journals further confirm the maturity of field research and form a clear “dual-track system for the dissemination of results.” On the one hand, Frontiers in Microbiology has become the largest platform for the rapid release and exchange of achievements in the field with a publication volume of 129 articles. Its high volume reflects the positioning of the journal as the dissemination and exchange of basic research results in the field. On the other hand, journals such as Microbiome and Nature Communications, although the volume of articles is limited, they have been cited with a high number of articles, establishing their position as the first choice carrier for top and highly influential research results in the field. This differentiation has formed an efficient communication system, which can meet the communication needs of research results at different stages and levels.

Analysis based on Bradford’s law shows that about 80% of the literature is published in about 15% of the core journals. This is not only a manifestation of the concentration of literature, but also means that these core journals have become the core communication platforms in this field, leading the discussion of key issues and knowledge evaluation standards. When selecting the target journal, the author actually chooses different communication positioning for the research results, or pursues extensive preliminary communication and discussion, or anchors the highest academic benchmark and influence. This clear journal hierarchy provides researchers with a clear strategic contribution map.

#### 4.1.4 Technology-driven paradigm leap and the evolution of success and failure of research topics

The evolution in this field is by no means a linear superposition of the number of literature, but a triple research paradigm leap driven by key technological breakthroughs. Each paradigm leap fundamentally reshapes the core scientific problems and knowledge output forms in the field, and is directly reflected in the rise and fall of research topics, clearly revealing the relative “success rate” and life cycle of different research directions.

The first paradigm (2005–2010): the completion of the outcome of “individual identification” and the basic theme under the cultivation of dependence. The end of the research at this stage is limited by traditional separation culture and one-generation sequencing technology, and the knowledge output is firmly limited to drawing scattered microbial “specimen atlas.” The core is to answer “what is it?” This basic problem (Reyes et al., 2010). Correspondingly, the keyword hotspots are concentrated on “*E. coli*,” “nucleotide sequence,” etc. These themes represent the foundational but completed stage tasks. Although their technical methods have been absorbed, they have been “retreated” as an independent research direction, reflecting the inevitable focus under the early technical constraints.

The second paradigm (2011–2018): the outcome of “community ecology” opened by macrogenomics and the outbreak of the mechanism theme. The popularity of macrogenome sequencing technology is a subversive turning point. It shifts the outcome of the research from individuals to communities, and the core question is upgraded to “How is the community composed and dynamically evolved?.” This paradigm leap directly gave rise to the first strategic shift of keywords: “diversity” and “horizontal gene transfer” entered the high outbreak period (Minot et al., 2011). This marks that the field has successfully entered the “golden age” of exploring community ecology and evolutionary dynamics from describing individuals. The representative result is to draw the first macro “ecological map” of the enterovirus group (Manrique et al., 2017). The success of these themes lies in systematically raising the research perspective to the complex system level.

The third paradigm (2019-present): the outcome of “functional intervention” and the rise of the application theme brought about by the integration of multiple studies. The progress of integrated analysis of single-viralomics, viral granulomics and multi-omics (Garmaeva et al., 2019) promotes the research focusing to directly refer to the health and clinical transformation of the host, aiming to answer “how does it affect the host? Can we regulate it accurately?” This is the ultimate problem (Federici et al., 2022; Federici et al., 2021). Under this paradigm, keyword hotspots achieve the final focus on “function” and “intervention.” Themes directly related to host metabolism, such as short-chain fatty acids, obesity, and thick-walled bacteria, continue to explode (Shkoporov and Hill, 2019), indicating that “microbiota functional metabolism and host disease interaction” has become a recognized and most potential frontier track. At the same time, the long-term popularity of “bacteriophage therapy” and “antibiotic resistance” (Strathdee et al., 2023) confirms the persistent pursuit of the field to solve practical clinical problems, which is the “beacon” direction of transformational research.

The cornerstone of success throughout: the theme of technology empowerment. It is worth noting that “bioinformatics,” as a key empowerment tool, has been hot for a long time (Shkoporov and Hill, 2019), highlighting the evergreen cornerstone of its evolution from an auxiliary means to a paradigm leap in the driving field. Its success reflects the fundamental dependence of research in this field on the computing power of complex data.

#### 4.1.5 Knowledge competition and inheritance in the co-citation network: verification and deepening of macro trends

The co-citation network analysis not only confirms the macro trend from “what is” to “how to regulate,” but more importantly, it reveals the underlying knowledge competition and inheritance relationship that supports this trend, that is, how different ideas, theories and methods interact to jointly promote the field forward.

##### 4.1.5.1 The formation of the foundational consensus: establishing the research object early highly cited literature

Such as Reyes et al. (2010) and Minot et al. (2011) constitute the most stable and basic core of the network. Their extensive and continuous citation marks a founding consensus in the academic community that “the enterovirus group is a real, complex and important research object.” This solves the problems of “legitimacy” and “descriptive framework” of research, and provides a common knowledge starting point for all subsequent explorations.

##### 4.1.5.2 Competition at the key turning point: from the theoretical focus of association to mechanism

Norman et al. (2015) with its strong explosive power and centrality, has become a key turning point in the network. It is not only a highly cited literature, but also a paradigm anchor to win in the knowledge competition-it has successfully directed the research focus from a broad “description” to a specific “disease mechanism.” Its success is that it provides a verifiable and extendable clear model (disease-viral group association), which has stimulated a large number of subsequent mechanism hypothesises and experimental designs.

##### 4.1.5.3 Contemporary frontier differentiation and collaboration: the double helix of theoretical deepening and tool innovation

Theoretical deepening cluster: For example, Shkoporov et al. (2019) focus on the construction of mechanism theories such as virus group stability and host interaction. Its close connection with disease-related literature shows that the field is trying to construct causal explanations for early “correlation” discoveries. Tool innovation cluster: for example, Nayfach et al. (2021) focus on developing virus identification, classification and analysis tools. Its strong connection with all theoretical clusters proves that the breakthrough of methodology has become the primary bottleneck and driving engine for unlocking new mechanisms and verifying new assumptions.

#### 4.1.6 Progress mode, evidence contradiction and transformation bottleneck in clinical research

The evolution trajectory of clinical research clearly reflects the ambition and challenges of moving from basic discovery to medical application in this field. A comprehensive analysis of 16 relevant studies reveals that a paradigm has been successfully transformed, but the evidence base is still facing a development stage of structural contradictions.

##### 4.1.6.1 Intervention from observation: the decisive success of the study paradigm

The most significant success of clinical research in this field is the completion of the paradigm leap from observation association to active intervention. The time distribution shows that between 2022–2024, the mean annual publication volume of interventional clinical studies (including RCT) surged to 2.7, an order of magnitude increase compared with the early stage (about 0.5 per year). This transformation marks that the field has gone beyond the primary stage of describing only the correlation between bacteriophage groups and diseases, and has officially entered a new era with the core goal of verifying its therapeutic and regulatory efficacy. Interventional research (RCT) accounts for 50%, which has become the mainstream path of current clinical exploration, reflecting the strategic shift of the focus of research.

##### 4.1.6.2 The “quality-scale” paradox of evidence production

At the level of evidence production, there is a contradiction pattern of “high rigor and low scale” that needs to be solved urgently. From a positive point of view, 14 of the 16 studies provide medium and high-level clinical evidence (mainly RCT and multi-center trials), indicating that the rigor of methodology has become a consensus in the field, which is an important normative achievement. But paradoxically, only 18.8% of them belong to large sample studies. The general small sample size constitutes the most prominent shortcoming of the current evidence system-the “small sample effect” may overestimate the intervention effect and seriously limit the statistical effectiveness and generalizability of the research results. This contradiction is essentially the gap between the requirements of “exploratory verification” and the final “certainty evidence” in the primary stage of clinical transformation. It is the core gap that the field must cross from “promising” to “proven.”

##### 4.1.6.3 “Concentration and imbalance” of disease focus

The distribution of research topics shows the characteristics of “high concentration and large blank space.” Inflammatory bowel disease (IBD) and metabolic-related diseases together constitute the “dual-core track” of the current clinical verification. This concentration stems from the clear association between these two types of diseases and the pathological mechanism of intestinal microbiota disorders, making it the most feasible “test site” to verify the concept. However, this focus has also led to a significant imbalance in the research map, and a large number of other areas of diseases closely related to intestinal microbiota (such as neuropsychiatric diseases, autoimmune diseases, etc.) still lack systematic interventional exploration. This imbalance is not only the result of the rational allocation of resources, but also may indicate a huge space for expansion in the future.

##### 4.1.6.4 Standardization of technical path and systematization of evaluation

At the methodological level, clinical research shows a high degree of technical convergence and evaluation maturity. 81.3% of the studies use macrogenomics as the core technology of viral group analysis, making it a gold standard tool for clinical research in this field, which ensures the potential comparability of data between different studies. It is worth mentioning that a complete three-level evaluation chain of “microbial structure–functional metabolism-clinical outcome” has become the mainstream paradigm. All studies monitored structural changes. 75% of the studies integrated the detection of functional metabolites (such as SCFAs and inflammatory factors), and more than 60% of the studies included direct clinical endpoint indicators. The wide adoption of this evaluation system marks that the field has a mature framework for the multi-dimensional effect of systematic evaluation interventions, which is an important manifestation of the depth of research.

In summary, this study systematically analyzes the academic development characteristics and evolutionary veins in the field of bacteriophage regulation of intestinal microbiota. The study found that the national level in this field has a differentiated development pattern, and institutional development and scholar cooperation have also formed a diversified success path. Open collaboration has become the core factor in enhancing academic influence; journal communication forms a dual-track system of “quick release” and “top results,” and the core journal group dominates knowledge exchange in the field and evaluation. The development of the field is driven by technological breakthroughs to complete three paradigm leaps, and gradually moves from the cultivation of dependent individual identification to the functional intervention of multi-group integration. Bioinformatics has become the key cornerstone of continuous empowerment. The co-citation network confirms the inheritance logic of field knowledge from establishing research objects to focusing on disease mechanisms and deepening theories and innovative tools. Clinical research has realized the paradigm shift from observation association to intervention verification. The methodological rigor and evaluation system are becoming more and more mature, but it still faces problems such as small sample size and unbalanced disease research layout, which has become the core bottleneck of clinical transformation in the field.

### 4.2 Future research prospects

#### 4.2.1 Mechanistic exploration: from description to elucidation

Most current studies still focus on describing the “correlation” between bacteriophages and the gut microbiota, but insufficiently elucidate the underlying specific “causality” and mechanisms of action. Future research should pay more attention to the in-depth exploration of the mechanism. On the one hand, it is necessary to strengthen the experimental verification of the interaction between bacteriophages and host bacteria. Currently, many predicted relationships rely on bioinformatics inferences from metagenomic data, whose accuracy is limited.

A more efficient experimental verification system needs to be developed in the future. For example, establish a high-throughput host-bacteriophage interaction screening platform, or use microflow control technology and synthetic biology to study the specificity and dynamic process of bacteriophage infection under conditions close to the physiological environment. This will help to define its ecological level more accurately (Džunková et al., 2019).

On the other hand, the specific mechanism of action of bacteriophages regulating the microbiota and affecting the health of the host has yet to be clarified. Although studies have shown that bacteriophages can affect metabolites such as short-chain fatty acids by changing the structure of the microbiota, their specific signaling pathways and effect molecules are still unclear (Duan et al., 2019). Integrating single-cell transcriptomics, spatial metabolomics and other multi-omics technologies, combined with *in vitro* models such as intestinal organs, will help to systematically describe the interaction network of “bacteriophage-microbiota-host health.”

#### 4.2.2 Clinical translation: from evidence accumulation to standardized application

Although early clinical studies have confirmed the potential of bacteriophage therapy, it still faces many practical challenges in its clinical application. At the level of research and design, it is urgent to promote rigorous clinical trials. It is recommended that the priority selection mechanism is relatively clear and there are indications that do not meet the clinical needs as a breakthrough, and a randomized controlled trial (RCT) with sufficient sample size should be carried out. The test plan should clarify the composition, route and course of treatment of the bacteriophage agent, and establish multiple endpoint indicators including clinical symptom improvement and changes in microbiota.

In terms of treatment strategies, the combined application of bacteriophages and traditional or emerging therapies can be explored. For example, bacteriophages and antibiotics can be considered to be used together or sequentially to reduce the risk of antibacterial drug resistance (Cao et al., 2015); or the two can be combined with prebiotics, probiotics and even fecal microbial transplantation (FMT) (Moye et al., 2018; Zuo et al., 2018), aiming to more effectively reconstruct the homeostasis of intestinal microorganisms through multi-target intervention (Hsu et al., 2019). In addition, with the deepening of the concept of personalized medical care, the development of “customized” mixed bacteriophage therapy based on the characteristics of the patient’s intestinal microbiota is also a promising development direction (Federici et al., 2022).

At present, the supervision of global bacteriophage agents has not yet formed a unified standard framework. In practical application, whether it is the establishment of the quality control system, the setting of safety assessment indicators, or the accurate definition of clinical indications, the relevant technical specifications and regulatory requirements are still in the stage of exploration and improvement. Against this background, promoting the deepening of cooperation between academia, industry, and regulatory departments and jointly formulating scientific and reasonable regulatory guidelines has become a key prerequisite for the transformation of bacteriophage therapy from basic laboratory research to clinical practice and finally the commercial application.

#### 4.2.3 Methodological innovation: from tool improvement to paradigm evolution

The progress of research methods is the fundamental driving force for expanding the cognitive boundaries of this field. At the level of experimental technology, it is urgent to develop a new method that can more accurately capture the activity and state of bacteriophages in the body. For example, the technology of developing the lysogenic and cleaving states (Dedrick et al., 2019), and the *in vivo* imaging method that realizes the dynamic real-time observation of bacteriophage infection in complex microbial communities (Díaz-Muñoz and Koskella, 2014), will significantly deepen our understanding of the ecological behavior of bacteriophages.

At present, due to the highly diverse and fragmented characteristics of the bacteriophage genome, the mainstream bioinformatics analysis process still has significant limitations in terms of detection sensitivity, taxonomy resolution and host prediction accuracy. In order to break through these bottlenecks, it is urgent to develop a new generation of algorithms. Specifically, it includes: (1) developing a high-sensitivity bacteriophage identification tool that does not rely on known reference sequences (Guo et al., 2021); (2) establishing a fine classification system that can effectively analyze its evolutionary trajectory (Turner et al., 2023); (3) integrating machine learning with multi-group signals to achieve higher-precision host association and functional annotation (Camargo et al., 2023). The breakthrough progress of these core algorithms is crucial to transforming massive sequencing data into reliable biological insights.

#### 4.2.4 Perspective expansion: diversified visions beyond human medicine

In the field of environmental science, bacteriophages are an important driving force for microbial evolution. Studying the interaction between bacteriophages and bacteria in different environments can deepen our understanding of the assembly mechanism of microbial communities (Koskella and Brockhurst, 2014).

From an evolutionary perspective, the study of bacteriophage-bacterial interaction helps to clarify the mechanism of microorganisms adapting to environmental changes and provides new insights into solving problems such as antibacterial drug resistance (Strathdee et al., 2023).

#### 4.2.5 Collaboration and ecosystem building: toward an open and inclusive scientific community

Meeting the above complex challenges requires the collaborative efforts of the global scientific community, and the continuous development and maintenance of high-quality open access to academic resource platforms is crucial. Improving the bacteriophage genome database by integrating more diverse sample data will help promote the overall development of this field (Roux et al., 2019).

Support should be provided for localization research in areas where research resources are relatively scarce. Different regions and populations have unique microbial compositions, and this diversity provides a valuable window for understanding microbial-environmental interactions (Thompson et al., 2017).

It is crucial to promote interdisciplinary exchanges and cooperation and integrate multi-field expertise. Regularly organizing academic exchange activities and forming interdisciplinary research teams will help produce innovative research results.

### 4.3 Research limitations

Although this study strives to be comprehensive, there are still some limitations. (1) Limited database coverage: Although the three major databases (WoSCC, Scopus and PubMed) are included, some high-quality research published in regional journals or unindexed literature may be omitted. In addition, the exclusion of non-English literature may introduce language bias. (2) Inherent limitations of literature measurement methods: Although citation-based analysis can reflect academic influence and knowledge flow, highly cited literature does not necessarily represent cutting-edge or innovative research. Keyword outbreak analysis and co-occurrence analysis are also limited by the degree of vocabulary standardization and the sensitivity of retrieval strategies. (3) The time limit for the inclusion of this research literature is until 2024, and there is a certain time lag effect on the academic dissemination and citation behavior of the thesis after it is published. Due to the short dissemination cycle, limited citation time, and low citations of literature recently published in 2024, their academic influence has not been effectively verified for enough time, which limits the interpretation effectiveness of the cited analysis results to a certain extent. Based on this, this study needs to carefully interpret the cited trend in this field, the disclosure of the context of research and development, and the prediction of related trends. (4) The depth of clinical literature analysis is limited: the discussion of clinical trials is mainly based on public abstracts and index descriptions, and the original research data is not obtained for quality evaluation. Therefore, the conclusion mainly reflects the strength of structural characteristics rather than the effectiveness evidence.

## 5 Conclusion

Through tools such as CiteSpace, VOSviewer and R-bibliometrix, this study systematically sorts out the academic picture in the field of bacterial phage regulation of intestinal microbiota from 2005 to 2024, and reveals the development path and ending of different academic entities through comparative analysis. The study found that the development of this field presents a clear technology-driven paradigm leap trajectory: from individual identification dependent on cultivation (2005–2010), to the analysis of community ecology opened by macrogenomics (2011–2018), to the function and intervention research under the current multi-group integration (2019-present). In the process, the research outcome has shifted from descriptive atlas to the mechanism exploration and targeted regulation of the complex interaction network of “bacteriophage-microbiota-host health.” The distribution of global research forces presents a differentiated pattern: the United States dominates with the model of “high output-broad cooperation-strong influence”; China has achieved a leading scale, but there is still room for improvement in the impact of results and the depth of international cooperation; Europe shows the path of “boutique-quality.” Core institutions and scholars have formed an interdisciplinary collaborative network with the University of Cork as the core, and have succeeded through the two modes of “continuous deep cultivation” and “precise breakthrough.” Research hotspots continue to evolve, and short-chain fatty acids, microbiota imbalances and clinical interventions have become the core frontiers, marking a comprehensive shift in the focus of the field to host health outcomes. Crucially, clinical translational research has entered a new stage of intervention verification and established a multi-dimensional evaluation framework of “structure-function-clinic.” However, its development is facing the core contradiction of “high-quality small-sample research” and “scarcity of large samples with low evidence intensity,” and the focus on diseases is too concentrated.

In summary, this field has made remarkable progress in revealing the key ecological and functional value of bacteriophages, but it still needs to overcome three bottlenecks to move toward clinical transformation: insufficient causal chain verification in the mechanism, lack of scale and breadth of clinical evidence, and gaps in the whole chain technical standards and supervision. Future research should focus on three directions: first, formulate a standardized framework for sample processing, data analysis, and clinical evaluation; second, combine multi-group technology and *in vitro* models to deepen the exploration of mechanism causal path; third, strengthen multi-center cross-regional collaboration, promote large-sample clinical trials, and promote the collaborative establishment of academic circles and regulatory authorities, so that the quality control system of bacteriophage agents accelerates the transformation from basic research to clinical practice in the field.

## Data Availability

Publicly available datasets were analyzed in this study. The names of the repositories, and details of the search methods used, can be found in the article. Further inquiries can be directed to the corresponding author.
